# Intracoronary Stenting and Restenosis – Randomized Trial of Drug-Eluting Stent Implantation or Drug-Coated Balloon Angioplasty According to Neointima Morphology in Drug-Eluting Stent REstenosis 5: Rationale and Design of the ISAR-DESIRE 5 Trial

**DOI:** 10.1007/s12265-025-10650-x

**Published:** 2025-06-30

**Authors:** Fiorenzo Simonetti, Felix Voll, Fernando Alfonso, Christian Gräßer, Marion Janisch, Michael Joner, Thorsten Kessler, Constantin Kuna, David Manuel Leistner, Tobias Lenz, Antonia Presch, Tobias Rheude, Hendrik Sager, Heribert Schunkert, Fabian Starnecker, Jens Wiebe, Adnan Kastrati, Salvatore Cassese, Erion Xhepa

**Affiliations:** 1 Klinik Für Herz- Und Kreislauferkrankungen, TUM Klinikum Deutsches Herzzentrum, Technische Universität München, Munich, Germany; 2 Department of Cardiology, Hospital Universitario de La Princesa, IIS-IP, CIVER-CV, Madrid, Spain; 3 DZHK (German Centre for Cardiovascular Research), Partner Site Munich Heart Alliance, Munich, Germany; 4 Universitäres Herz- Und Gefäßzentrum, Universitätsklinikum Frankfurt, Frankfurt, Germany; 5 Deutsches Zentrum Für Herz-Kreislauf-Forschung, Munich, Germany

**Keywords:** Drug-coated balloon, Drug-eluting stent, In-stent restenosis, Optical coherence tomography, Percutaneous coronary intervention

## Abstract

In-stent restenosis (ISR) is the leading cause of revascularization failure, occurring in up to 10% of patients within 10 years after drug-eluting stent (DES) implantation, and is associated with increased mortality and rehospitalization. Guideline-writing authorities recommend DES over drug-coated balloons (DCB) for ISR treatment. However, this indication is mainly based on trials that did not incorporate intravascular imaging. Recent findings suggest that optical coherence tomography (OCT) patterns in ISR may influence treatment outcomes. The ISAR-DESIRE 5 trial is a randomized study to evaluate whether OCT-defined ISR tissue morphology affects treatment with DES versus DCB. 376 patients with ISR will be stratified by OCT pattern (homogeneous vs. non-homogeneous) and randomized 1:1 to DES or DCB. The trial is powered to detect an interaction between OCT pattern and treatment modality on the 24-month incidence of major adverse cardiac events and represents a step toward establishing a more individualized approach to ISR management.

**Trial registration (ClinicalTrials.gov):** NCT05544864.

**Graphical Abstract:**

ISAR-DESIRE 5 Trial design overview. After classification of the predominant OCT restenotic pattern at the site of the MLA as either homogeneous or non-homogeneous, patients will undergo 1:1 randomization to receive either a DES or a DCB, using a stratified randomization approach. This unique design ensures balanced allocation of treatment modalities (DES or DCB) across the two OCT-defined restenotic patterns. DCB: Drug-Coated Balloon; DES: Drug-Eluting Stent; MLA: Minimum Lumen Area; OCT: Optical Coherence Tomography

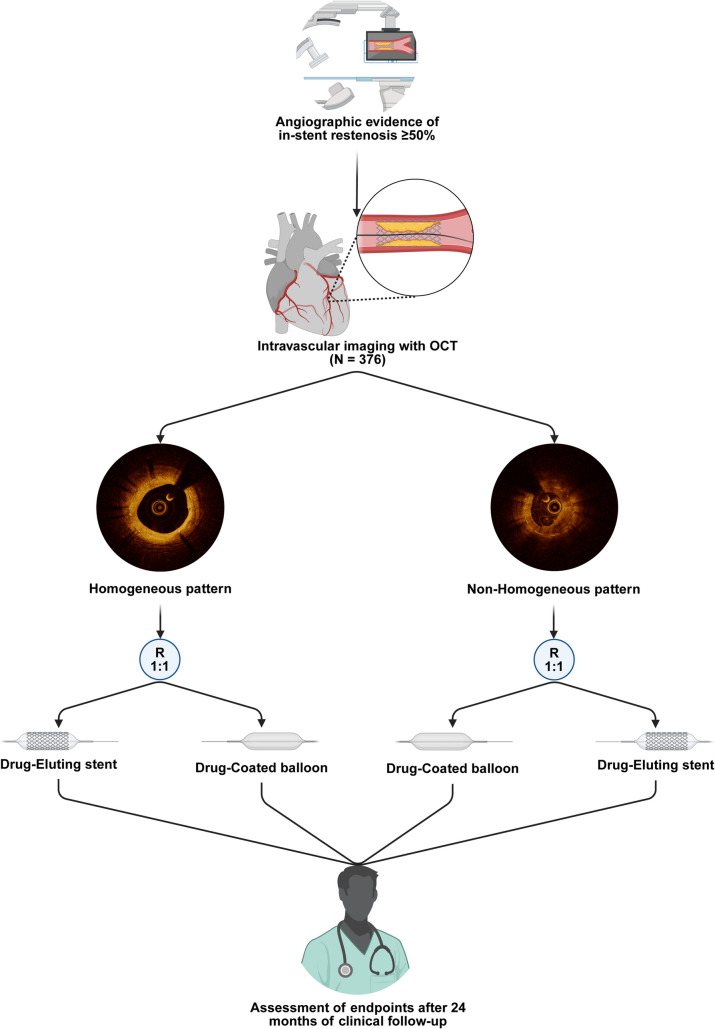

**Supplementary Information:**

The online version contains supplementary material available at 10.1007/s12265-025-10650-x.

## Introduction

Despite significant advances in the percutaneous treatment of coronary artery disease (CAD), in-stent restenosis (ISR) remains the most common cause of revascularization failure following percutaneous coronary interventions (PCI) [1].

Indeed, although significantly less frequent than after bare-metal stent (BMS) implantation, the incidence of clinical drug-eluting stent (DES) ISR is up to 10% within the first 10 years after DES implantation [2].

ISR is strongly associated with patient and lesion complexity, and the proportion of complex patients has steadily increased over the years [3]. Hence, despite the improvements in PCI technology, the proportion of ISR is expected to increase in the coming years. This proportion, combined with the very high number of DES implanted around the world and its association with an increased risk of death and rehospitalization [3–5] makes ISR a clinical entity of strong public health significance [6].

In the last decade, with the advent of new-generation DES, this longstanding issue was believed to have been definitively solved. Indeed, randomized clinical trials have consistently documented a significant decrease in the risk of repeat revascularization with modern DES compared with earlier platforms [7–12]. Nevertheless, ISR continues to represent a clinical conundrum, since each intervention for ISR portends a higher risk for subsequent reintervention, with an increasing risk of recurrent ISR after treatment with DES or drug-coated balloons (DCB), particularly in presence of 3 metal layers [13, 14].

Several strategies for the treatment of ISR have been tested over the years [15], but DCB angioplasty and repeat DES implantation have emerged as the most effective options [16–20].

Guideline-writing authorities recommend DES over DCB for ISR treatment (Class I, Level A), mainly based on meta-analyses of trials published between 2013 and 2017, focusing on relative risk of recurrent revascularization [21]. Although expert guidelines support the use of intravascular imaging (IVI) for the management of ISR, a common limitation of all trials comparing treatments for ISR is the lack of systematic IVI assessment to evaluate potential advantages and/or drawbacks of different treatment strategies in specific IVI-defined ISR subsets.

As a result, it is still unclear how IVI findings may influence ISR treatment strategies.

**Fig. 1:**
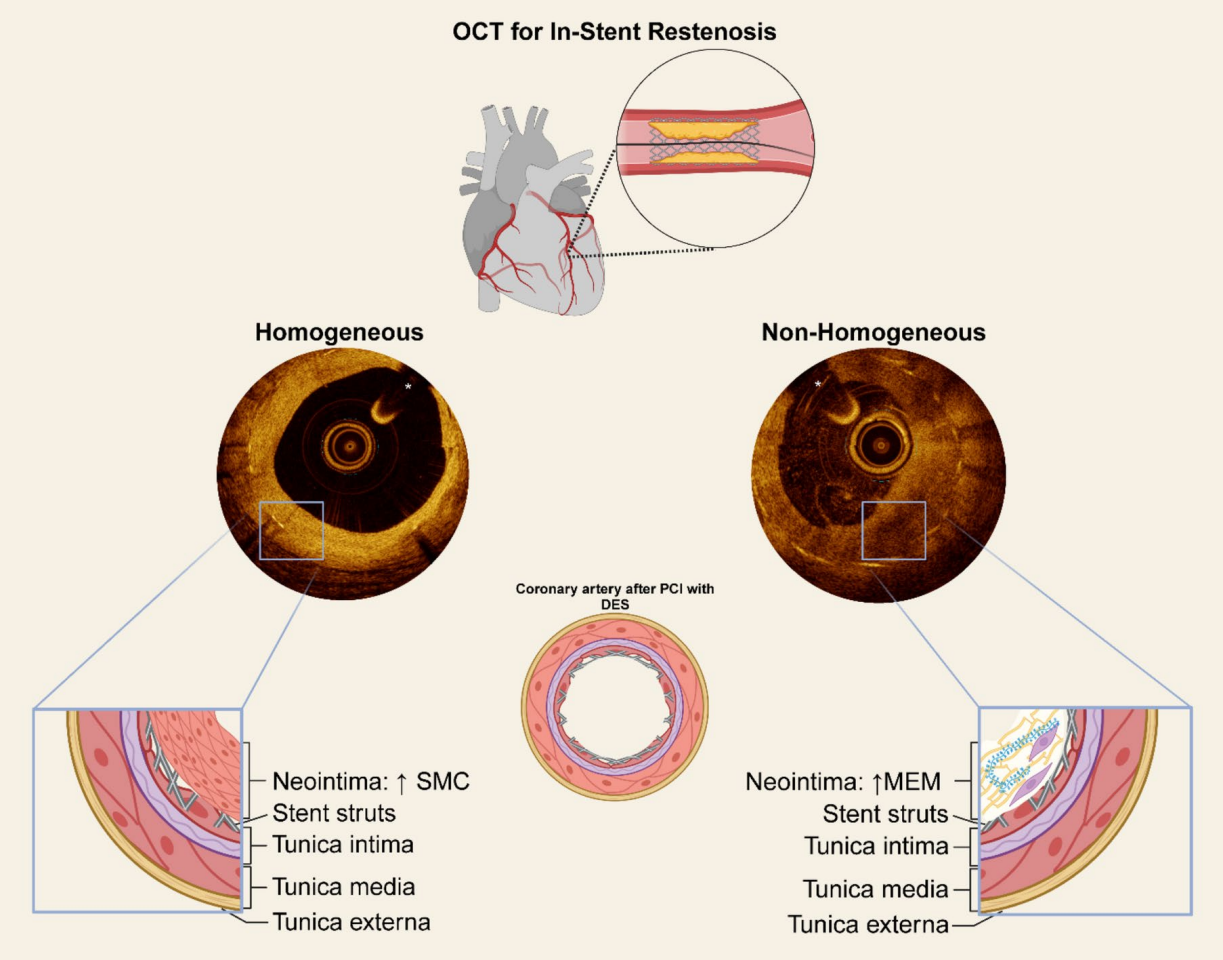
Patterns of In-Stent Restenosis on Intravascular Optical Coherence Tomography and Corresponding Histological Substrates in the ISAR-DESIRE 5 trial. Tissue characterization will be performed at the frame of minimum lumen area. Based on its optical properties at intravascular OCT imaging, restenotic tissue has been subdivided into several patterns that correlate with different histological substrates. Previous histopathological validation studies of intravascular OCT findings have shown homogeneous patterns to consistently correlate with abundance of smooth muscle cells, whereas the remaining patterns are typically characterized by a multitude of histological findings. Aiming to apply a pragmatic, clinically oriented and easily applicable algorithm, restenotic tissue will be categorized as predominantly homogenous or non-homogenous. The latter category includes frame with prevalent heterogeneous, layered aspect and/or with neo-atherosclerosis. *: guidewire artifact. DES: Drug-Eluting Stent; MEM: Myxomatous Extracellular Matrix; OCT: Optical Coherence Tomography; SMC: Smooth Muscle Cells

Particularly, high-resolution IVI by means of intravascular optical coherence tomography (OCT) provides unique insights into the mechanisms leading to the occurrence of ISR, as well as the characteristics of restenotic tissue. According to such optical characteristics, restenotic tissue can be classified into several patterns, which have been shown to correlate with different histological substrates (Fig. 1) [22–24].

These differing tissue patterns may impact the clinical outcomes of patients undergoing PCI for ISR in a way that is dependent on the treatment method (DES or DCB). However, the number of studies investigating such an hypothesis is very scarce, and to date, no prospective, randomized clinical data are available [25].

Against this background, this study aims to investigate the interaction between different OCT-defined tissue patterns and the relative efficacy of different interventional strategies (DES vs. DCB) in patients with ISR in a prospective, randomized fashion.

## Methods

Intracoronary Stenting And Restenosis – randomized trial of Drug-Eluting Stent Implantation or drug-coated balloon angioplasty according to neointima morphology in drug-eluting stent REstenosis 5 (ISAR-DESIRE 5, ClinicalTrials.gov: NCT05544864) is an investigator-initiated, multicenter, prospective, stratified, randomized controlled trial (RCT), designed to investigate a potential interaction between different restenotic tissue patterns and treatment modality (DCB or DES) of ISR lesions in patients presenting with DES-ISR.

The hypothesis being tested is that a significant interaction between OCT-defined tissue patterns (homogeneous or non-homogeneous) and treatment modality with the currently recommended ISR treatment strategies (DES or DCB) exists.

A total of 376 patients will be prospectively enrolled in Germany, Spain, and Belgium. The study started in September 2022 and is expected to conclude in 2026.

This trial will be conducted in accordance with the Declaration of Helsinki, ISO 14155, and applicable local laws and regulations. The protocol, amendments, and the subject's informed consent were approved by all participating sites'independent ethics committees (IEC).

Before enrollment in the study, investigators will inform patients orally and in writing about the scope, purpose, and possible risks/benefits of the study and his/her rights and duties in lay language. Signed informed consent must be collected before randomization. All patient data will be protected and anonymized by a specific identification code and entered into an electronic case reporting form (eCRF).

### Study Population

Patients ≥ 18 years old who have signed the written informed consent for participation in the study will be considered eligible for inclusion if they have ischemic symptoms and/or evidence of myocardial ischemia (including non-invasive or invasive functional tests), angiographic evidence of ≥ 50% diameter stenosis (visual estimation) in a native coronary vessel previously treated with DES, as well as availability of an OCT pullback of the target lesion. Key exclusion criteria are represented by cardiogenic shock, acute ST-elevation myocardial infarction (MI) within 48 h from symptoms onset, target lesion location in the left main coronary artery or in a bypass graft, or unsuccessful treatment of other lesion(s) during the same procedure. Multiple stent layers will not be considered an exclusion criterion. The complete list of inclusion and exclusion criteria is shown in Table 1.

**Table 1:** Complete list of inclusion and exclusion criteria

**Inclusion criteria**
1) Age ≥ 18 years
2) Patients with ischemic symptoms and/or evidence of myocardial ischemia
3) Presence of ≥ 50% angiographic restenosis (visual estimation) after prior implantation of drug-eluting stents in native coronary vessels
4) Availability of an OCT-pullback of the target lesion
5) Written informed consent by the patient for participation in the study
**Exclusion criteria**
1) Cardiogenic shock
2) Acute ST-elevation myocardial infarction within 48 h from symptom onset
3) Target lesion located in left main or bypass graft
4) Additional coronary intervention planned within 30 days of the procedure
5) Non-successful treatment of other lesion(s) during the same procedure
6) Severe kidney disease (eGFR ≤ 30 ml/min/m^2^)
7) Contraindications to any components of the investigational devices or dual antiplatelet therapy
8) Pregnancy (present, suspected, or planned) or positive pregnancy test
9) Previous enrollment in the same trial or participation in any other study at the time of enrollment
10) Malignancies or other comorbidity conditions with a life expectancy of less than 12 months or that may result in protocol non-compliance
11) Patient’s inability to fully comply with the study protocol

*eGFR* estimated Glomerular Filtration Rate, *OCT* Optical Coherence Tomography

### Randomization and Study Procedures

Patients meeting all inclusion and none of the exclusion criteria will be considered eligible for participation in the study. Eligible patients will be randomized using an electronic randomization system in the chronological order in which they qualify. Patients will first be characterized during the procedure according to the OCT-defined restenotic pattern (homogeneous or non-homogeneous). Then, they will be randomized to either DES or DCB treatment in a 1:1 stratified way based on OCT-defined restenotic tissue in order to obtain an equal distribution of treatment modality within OCT patterns (Graphical Abstract). Patients randomized to repeat DES implantation will be treated with a permanent-polymer everolimus-eluting stent (Xience, Abbott Vascular), while those randomized to DCB angioplasty will undergo treatment with any CE-approved, commercially available DCB. Particular care will be paid to ensure optimal lesion preparation. It is strongly recommended to consider OCT findings when determining the PCI strategy (e.g.: choice of device size and length) (Supplemental Methods S1).

**Fig. 2:**
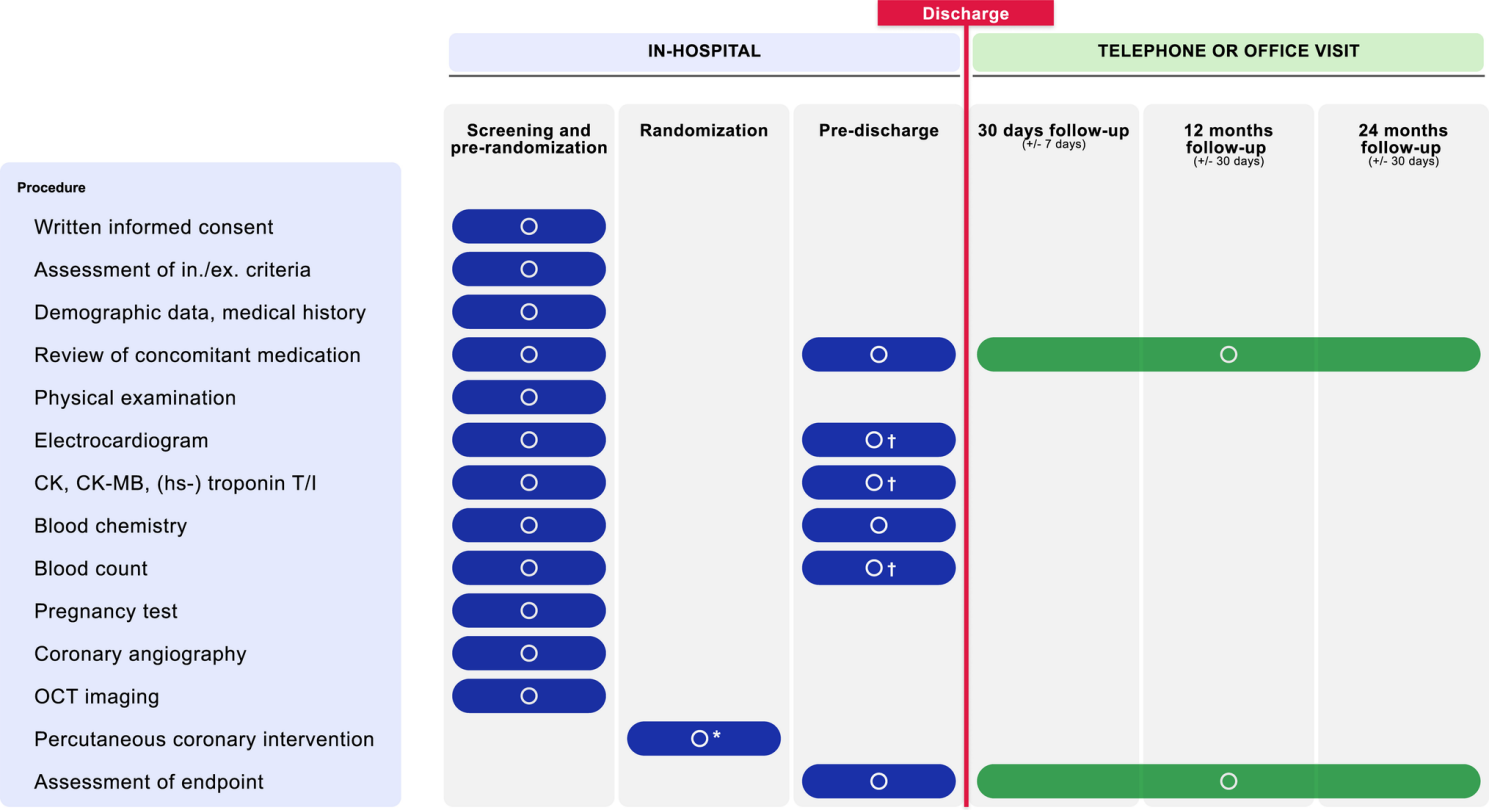
Scheduled activities during different periods of the ISAR-DESIRE 5 Trial. *: PCI will be performed only after randomization. †: Within 24 h after randomization. Hs: High sensitivity; OCT: Optical Coherence Tomography; PCI: Percutaneous Coronary Intervention

Additional lesions must be treated before the study lesion. In the case of multiple ISR lesions, the highest-grade ISR will represent the target lesion. Baseline clinical and interventional procedures will be performed according to standard clinical practice before randomization (Supplemental Methods S2 and Fig. 2).

In the case of sub-occlusive ISR, precluding sufficient blood clearance through contrast medium and insufficient OCT image quality, lesion predilation with a 1.5 mm balloon will be performed to allow adequate vessel opacification and optimal OCT image quality.

A comprehensive description of peri-interventional and post-interventional therapies is provided in the Supplemental Methods S3.

### OCT Assessment

Real-time assessment of the intravascular OCT pullback will be performed by the operator in order to confirm the presence of ISR at the target lesion as well as to conduct qualitative characterization of restenotic tissue at the site of minimum luminal area (MLA) within the restenotic segment, which will ultimately guide stratification during the randomization process [25, 26].

The restenotic pattern will be defined as predominantly homogeneous (≥ 50% uniform optical proprieties that don’t show any focal variation in backscattering patterns) or non-homogeneous (focally changing optical properties with various backscattering patterns or a layered appearance) (Fig. 1). This classification was adapted from that originally proposed by Gonzalo et al. [24]. Detailed definitions are provided in the Supplemental Methods S4. Based on its optical properties at intravascular OCT imaging, restenotic tissue has been subdivided into several patterns that correlate with different histological substrates. Previous histopathological validation studies of intravascular OCT findings have shown homogeneous patterns to consistently correlate with an abundance of smooth muscle cells. In contrast, the remaining neointimal patterns are typically characterized by a multitude of histological findings, including myxomatous extracellular matrix, proteoglycans, organized fibrin and neoatherosclerosis [25–27]. Based on the findings of the aforementioned OCT-histology correlation studies and aiming to apply a pragmatic, clinically oriented, and easily applicable algorithm, restenotic tissue will be categorized as predominantly homogenous or non-homogenous.

Subsequently, a detailed offline analysis of OCT pullbacks, including morphometric measurements and qualitative tissue characterization, will be performed in a blinded way at the ISAResearch Center (German Heart Center, Munich, Germany), which will serve as the core laboratory for the present trial (Supplemental Figure S1). Following the enrollment of the first few patients, the core laboratory will provide feedback to participating centers on the qualitative tissue characterization.

### Patient Follow-up

Patients will be prospectively clinically monitored for endpoint-relevant clinical events throughout the study period. Furthermore, the concomitant medication will be reviewed. Patients will be clinically monitored daily during their in-hospital stay and contacted by phone or visited in-office at 30 ± 7 days, 12 months ± 30 days, and 24 months ± 30 days (Fig. 2).

### Study Endpoints

The primary endpoint of the study is represented by the composite of major adverse cardiac events (MACE) defined as all-cause death, MI, and target lesion revascularization (TLR) after 24 months of clinical follow-up. The prespecified secondary endpoints are represented by the individual components of the primary composite endpoint, target lesion failure (TLF), defined as a composite of cardiac death, target vessel MI and TLR, stent thrombosis (ST) according to the academic research consortium (ARC) criteria as well as the composite safety endpoint represented by of all-cause death and MI. Detailed definitions of the single endpoint components are provided in the Supplemental Methods S5.

### Statistical Analysis and Sample Size Calculation

According to current evidence, the incidence of homogeneous and non-homogeneous ISR patterns is comparable [25, 26], and thus, both arms are sized equally. We used a z-Test for the interaction effect of homogeneous morphology (yes/no) and stent treatment (yes/no) in a proportional hazard regression model. Based on the results of our previous explorative observational analysis [27], we assume a proportion of primary endpoint events of 12.5% in the group with non-homogeneous morphology and PCI with DES, of 40% in the group with non-homogeneous morphology and PCI with DCB and 27% in both groups (treated with DCB or DES) with homogenous morphology. With a power of 80%, a two-sided 5% significance level, and assuming a 25% drop-out due to patients lost to follow-up, the total estimated sample size of the study is 376 patients.

The analysis will be based on the intention-to-treat (ITT) population. The full analysis set (FAS) consists of all randomized patients. According to the ITT principle analysis, the FAS will allocate patients to treatment groups as randomized. The per-protocol (PP) population includes all patients of the FAS without protocol deviations. The primary endpoint will be analyzed using a Cox proportional hazards regression model, including the interaction effect of restenotic morphology (homogeneous vs. non-homogeneous) and treatment strategy (DES vs. DCB). Both factors will be included as binary (dummy-coded) variables. This approach will allow assessment of whether the effect of treatment differs according to the underlying OCT-defined restenotic morphology. The exploratory hypothesis of the primary and secondary endpoints will be performed similarly to the confirmatory analysis of the primary endpoint using ITT and PP populations. Event-times distribution will be estimated using the Kaplan–Meier method and tested for group differences by exploratory long-rank tests.

Descriptive statistics will be computed for baseline variables as appropriate.

Prespecified subgroup analysis will be performed for each of the following subgroups: age, sex, body mass index, diabetes mellitus, renal function, clinical presentation, angiographic classification of ISR, vessel size, and use of advanced lesion preparation devices (modified balloon, super-high pressure balloon, intravascular lithotripsy, and atheroablative techniques). Results will also be analyzed according to the OCT tissue appearance according to the central core lab analysis.

### Study Organization

The ISAR-DESIRE 5 Trial is an investigator-initiated trial financed by the Munich German Heart Centre and additionally supported by an unrestricted educational grant from Abbott Vascular. The steering committee is responsible for overseeing the good execution and administrative progress of the protocol. The clinical trial documents will be made available by the principal investigator upon request by monitors, auditors, and other representatives of the sponsor or authorities, provided that the data are kept confidential and the patient’s privacy is guaranteed.

### Clinical Event Adjudication Committee

The Clinical Event Committee (CEC) will review and decide on all clinical endpoints. The CEC will create clear rules and processes for classifying a clinical event, using specific criteria based on the definitions in the study protocol. All committee members will be blinded to the randomized treatment assignments for all adjudicated patients.

## Discussion

The ISAR-DESIRE 5 trial is the first prospective, randomized clinical trial designed to test the hypothesis of a significant interaction between OCT-defined tissue pattern of ISR lesions (homogenous or non-homogeneous) and treatment effect of DES vs. DCB in terms of a composite primary endpoint (MACE) at 24 months follow-up.

Several prospective, randomized clinical trials have already analyzed the clinical outcomes of patients undergoing DES or DCB treatment for ISR lesions, with partially mixed results. However, an important limitation of virtually all randomized trials comparing treatment strategies for ISR lesions is the lack of systematic IVI assessment to evaluate potential differences in clinical outcomes based on the neointimal characteristics of ISR subsets.

In the BIOLUX – Clinical performance of the Pantera LUX paclitaxel releasing balloon versus the drug-eluting Orsiro hybrid stent system in patients with in-stent restenosis (BIOLUX) trial [17], Jensen et al. found comparable results in terms of late lumen loss (LLL) (DCB vs. DES Δ = −0.17 ± 0.52 mm, 97.5% CI; p non-inferiority < 0.0001) and TLF (DCB vs. DES: 17.9% vs. 18.6%, p > 0.99) between DCB and DES treatment strategies. Compared to the present trial, this trial had a shorter follow-up period (18 months), and IVI was not mandatory; therefore, it did not provide conclusive evidence on the effects of either treatment modality.

In the Intracoronary Stenting and Angiographic Results: Drug Eluting Stent In-Stent Restenosis: 3 Treatment Approaches (ISAR-DESIRE 3) [18], Byrne and colleagues tested the non-inferiority of paclitaxel-eluting balloon (PEB) compared with paclitaxel-eluting stent (PES) and the superiority of both PEB and PES compared with balloon angioplasty alone. PEB was shown to be non-inferior to PES in terms of diameter stenosis (38.0% [SD 21.5] vs 37.4% [21.8]; difference 0.6%, one-sided 95% CI 4.9%; p non-inferiority = 0·007; non-inferiority margin of 7%). Some important limitations, mainly represented by the use of a surrogate angiographic endpoint, first generation DES platforms as well as the lack of IVI guidance, limit the applicability of these findings to the current clinical practice.

The more recent Restenosis Intra-Stent of Drug-Eluting Stents: Drug-Eluting Balloons vs Everolimus-eluting Stents (RIBS IV) [19] trial aimed at addressing these issues by performing a comparison of long-term (3 years) clinical outcomes of patients undergoing ISR treatment by means of DEB or everolimus-eluting stents. The 3-year clinical follow-up of this randomized clinical trial showed that in patients presenting with DES-ISR, everolimus-eluting stents reduce the need for repeat interventions compared with DEB. However, besides the lack of IVI guidance, a further limitation of this study is represented by the lack of statistical power to discern differences in clinical endpoints, which notably constrains the value of various sub-analyses.

Of note, regardless of the treatment strategy adopted, the overall risk of MACE remains constantly higher in patients undergoing PCI for ISR as compared to those undergoing PCI for de novo coronary lesions, highlighting the need for individualized treatment strategies in this patient population.

In recent years, several observational studies applying intravascular OCT sought to perform a detailed characterization of the substrates underlying ISR lesions as well as to investigate the clinical outcomes of differing lesion subsets. From a pathological standpoint, while ISR following BMS implantation is characterized by neointimal hyperplasia consisting mainly of high proportions of smooth muscle cells, ISR following DES implantation is characterized by a more mixed appearance, including proteoglycan-rich neointimal hyperplasia and lesser amounts of smooth muscle cells [1]. Additionally, the development of neoatherosclerosis, defined as de-novo atherosclerotic plaque within a previously stented segment, has been shown to be a time-dependent phenomenon which tends to occur earlier and more frequently following DES as compared to BMS [1, 28–30].

Previous OCT-histology validation studies of intravascular OCT findings have shown homogeneous neointimal patterns to consistently correlate with abundance of smooth muscle cells, which are thought to display high proliferative activity, whereas the remaining restenotic tissue patterns are typically characterized by a multitude of histological findings [22, 25–27].

The action mechanism of DCB relies on rapid initial transfer and subsequent tissue retention of an antiproliferative agent necessary for persistent suppression of cell proliferation. Such an action mechanism suggests that the subset of smooth muscle cell-rich ISR lesions (homogeneous neointima at OCT imaging) might represent a particularly suitable substrate for DCB treatment. If favorable clinical outcomes in this lesion subset are confirmed, DCB could be potentially able to combine the avoidance of additional stent layers with optimal clinical outcomes. Indeed, although clinical outcomes following DES implantation might be less dependent on underlying restenotic tissue substrate compared to DCB, repeat DES implantation has potential drawbacks, represented mainly by the presence of additional stent layers and accelerated neo-atherosclerosis development, which could trigger late adverse events [28].

According to these assumptions, Tada et al. found in a preliminary explorative analysis on 428 PCI for ISR lesions that recurrent ISR and TLR rates were significantly higher in homogeneous pattern with plain old balloon angioplasty (POBA) than in DCB group (ISR: 54.8% vs. 19.1%, p < 0.001; TLR: 38.7% vs. 10.6%, p < 0.001) and DES group (ISR: 54.8% vs. 19.6%, p = 0.002; TLR: 38.7% vs. 10.7%, p = 0.005), whereas no differences were detected in ISR and TLR rates in lesions with heterogeneous structures [25].

In addition, a more recent analysis, which sought to investigate the relationship between OCT-defined restenotic tissue pattern (homogeneous vs. non-homogeneous) and treatment modality of ISR (DCB vs. DES) in terms of hard clinical endpoint (MACE), showed a significant advantage of DES over DCB in the non-homogeneous lesion subset (MACE: HR 0.023 [0.10–0.65], p = 0.004; TLR: HR 0.28 [0.11–0.69], p = 0.006), while the two treatment strategies were associated with comparable clinical outcomes in the homogeneous subset of ISR lesions, suggesting that a significant interaction between neointimal pattern and treatments modality exist [27].

Against this background, the ISAR-DESIRE 5 trial was designed to test this hypothesis in a prospective, randomized setting. To the best of our knowledge, several trials are currently exploring treatment modalities for ISR, but none has a similar profile (Supplemental Table S1).

A potential limitation of this approach is the generalizability of OCT assessment of restenotic tissue patterns, particularly in centers without routine experience in OCT interpretation. Although inter-operator variability in image interpretation may exist, current guidelines and growing randomized evidence increasingly support the use of (including OCT) in the routine practice. Furthermore, our study applies a classification based solely on the identification of predominantly homogeneous or non-homogeneous patterns, rather than the full spectrum of subtypes previously proposed [24], thus reducing the learning curve and increasing the broader clinical applicability.

In conclusion the ISAR-DESIRE 5 is the first prospective, randomized, controlled, multicenter trial testing the hypothesis of an interaction between OCT-defined restenotic tissue pattern and treatment modality (DCB vs. DES) of ISR. Although several randomized trials have sought to compare DCB and DES for the treatment of ISR, representing the basis for the current guideline recommendations, a common limitation of available trials is represented by the lack of systematic IVI to evaluate potential differences in clinical outcomes between different restenotic tissue subsets. Consequently, the guiding role of intravascular OCT and the potential interaction between underlying tissue patterns and treatment modality of ISR have not been explored in a randomized setting. Based on preliminary evidence from observational data, the ISAR-DESIRE 5 randomized clinical trial aims to investigate the presence of such an interaction in a prospective, randomized fashion, thereby expanding knowledge in this evidence gap.

## Clinical Relevance

In-stent restenosis (ISR) continues to be the leading cause of revascularization failure, despite advances in stent technology. While intravascular imaging, particularly optical coherence tomography (OCT), is increasingly used in clinical practice, its potential to guide ISR treatment strategies remains poorly defined. Emerging data suggest that different OCT-defined restenotic patterns may influence the effect of drug-eluting stent (DES) implantation or drug-coated balloon (DCB) percutaneous coronary intervention (PCI) in ISR, yet clinical evidence supporting a tailored, imaging-guided approach is limited. The ISAR-DESIRE 5 trial will address this unmet need by evaluating whether OCT-defined restenotic tissue characteristics influence the clinical outcomes of patients treated with DES or DCB. This study represents a critical step toward establishing a more individualized and evidence-based approach to ISR management.

## Supplementary Information

Below is the link to the electronic supplementary material.

Supplementary file1 (DOCX 496 KB)

## Data Availability

The documents of the clinical trial must be made available upon request by monitors, auditors, and other representatives of the sponsor or authorities, provided that the data are kept confidential and the patient’s privacy is guaranteed.

## Abbreviations

ARC: Academic Research Consortium
BMS: Bare Metal Stent
CAD: Coronary Artery Disease
CEC: Clinical Event Committee
DCB: Drug-Coated Balloon
DES: Drug-Eluting Stent
eCRF: Electronic Case Reporting Form
ESC: European Society of Cardiology
FAS: Full Analysis Set
IEC: Independent Ethics Committees
ISR: In-Stent Restenosis
ITT: Intention-To-Treat
IVI: Intravascular Imaging
LLL: Late Lumen Loss
MACE: Major Adverse Cardiac Events
MI: Myocardial Infarction
MLA: Minimum Lumen Area
OCT: Optical Coherence Tomography
PCI: Percutaneous Coronary Intervention
PEB: Paclitaxel Eluting Balloon
PES: Paclitaxel Eluting Sent
POBA: Plain Old Balloon Angioplasty
PP: Per Protocol
RCT: Randomized Controlled Trial
ST: Stent Thrombosis
TLF: Target Lesion Failure
TLR: Target Lesion Revascularization
