# Supplementary material for: Intracoronary Stenting and Restenosis – Randomized Trial of Drug-Eluting Stent Implantation or Drug-Coated Balloon Angioplasty According to Neointima Morphology in Drug-Eluting Stent REstenosis 5: Rationale and Design of the ISAR-DESIRE 5 Trial
Source: J Cardiovasc Transl Res. 2025 Jun 30;18(5):1176–84. doi: 10.1007/s12265-025-10650-x (PMC12630265; doi:10.1007/s12265-025-10650-x)
Supplement: Supplementary file 1 — Supplementary file1 (DOCX 496 KB) [file 12265_2025_10650_MOESM1_ESM.docx]

**Supplementary Material**

**Intracoronary Stenting And Restenosis – randomized trial of Drug-Eluting Stent Implantation or drug-coated balloon angioplasty according to neointima morphology in drug-eluting stent REstenosis 5: Rationale and Design of the ISAR-DESIRE 5 Trial**

[Supplemental Tables 2](#_Toc195895029)

[Supplemental Table S1. Main characteristics of currently ongoing randomized trials investigating interventional strategy for In-stent restenosis. 2](#_Toc195895030)

[Supplemental Figures 4](#_Toc195895031)

[Supplemental Figure S1. Representative Core Lab OCT Analysis from the ISAR-DESIRE 5 Trial. 4](#_Toc195895032)

[Supplemental Methods 5](#_Toc195895033)

[Supplemental Methods S1. OCT-derived PCI-guidance 5](#_Toc195895034)

[Supplemental Methods S2. Baseline clinical and interventional procedures performed according to standard clinical practice before randomization in the ISAR-DESIRE 5 Trial 6](#_Toc195895035)

[Supplemental Methods S3. Peri and post-interventional therapy in the ISAR-DESIRE 5 trial 7](#_Toc195895036)

[Supplemental Methods S4. Detailed definitions of qualitative OCT assessment 8](#_Toc195895037)

[Supplemental Methods S5. Endpoint’s definitions of the ISAR-DESIRE 5 trial 9](#_Toc195895038)

# Supplemental Tables

## Supplemental Table S1. Main characteristics of currently ongoing randomized trials investigating interventional strategy for In-stent restenosis.

| Trial | Description | Primary endpoint | Arms | Patient N |
| --- | --- | --- | --- | --- |
| MAGICAL ISR (NCT05908331) | A Prospective, Multicenter, Randomized, Two-Arm, Single-blind Superiority Trial to Evaluate the Safety and Efficacy of the MagicTouch™ Sirolimus- Coated Balloon in the Treatment of Coronary DES ISR. | TLF [Time Frame: 12 months]:  The composite rate of cardiac death, target-vessel MI or ischemia-driven TLR | **Experimental:** Sirolimus Drug Coated Balloon MagicTouch™  **Control:** POBA | 492 |
| ISAR-DESIRE 5  (NCT05544864) | A Prospective, Multicenter, Randomized Trial with the hypothesis of the study that there is a significant interaction in treatment effect between the OCT pattern of neointima (heterogeneous or homogeneous) and the type of percutaneous coronary intervention (DES or DCB) in patients with ISR. | Composite endpoint of MACE [Time Frame: 24 months of clinical follow-up after randomization]: all-cause death, MI or TLR | **Experimental:** Homogenous: DES vs. DEB  **Control:** Heterogeneous: DES vs. DEB | 376 |
| OPEN-ISR  (NCT04862052) | An open label, randomized study to compare the safety and efficacy of three different methods to handle coronary DES ISR. | - Occurrence of new onset target vessel myocardial infarction [Time Frame: 6 months]: New onset MI that affects the target vessel treated with either study device; - Occurrence of target vessel revascularization of failure [Time Frame: 6 months]: The need for further revascularization affecting the target vessel treated with either study device, or the failure of the vessel treated by either study device; - Occurrence of TLR [Time Frame: 6 months]: The need for further revascularization of the target lesion after treatment with either study device. | **Experimental:** Sirolimus Drug Coated Balloon MagicTouch™  **Experimental:** Paclitaxel coated balloon Emperor  **Control:** Everolimus eluting stent Xience | 150 |
| SELUTION4ISR  (NCT04280029) | A Prospective Randomized Single Blind Multicenter Study to Assess the Safety and Effectiveness of the SELUTION SLR™ 014 Drug Eluting Balloon in the Treatment of Subjects With ISR | TLF [Time Frame: 12 months post-index procedure]: all cardiac death, target vessel MI or clinically driven TLF. MI includes spontaneous (Type 1) MI using the 4th UDMI and peri-procedural MI using the SCAI definition. | **Experimental:** SELUTION SLR™ DEB  **Control:** POBA or FDA-approved -limus DES | 392 |
| UNIQUE-DCB-II  (NCT04119986) | A multicenter, randomized and prospective study aiming to evaluate the safety and efficacy of drug balloon therapy for ISR in patients with CHD under the guidance of QFR compared with DES implantation. | The incidence rate of LLL (evaluated by QCA) after PCI in patients with ISR [Time Frame: Follow-up coronary angiography at 12 months after the procedure] | **Experimental:** DCB  **Control:** DES | 220 |
| REFORM  (NCT04079192) | A prospective, multi-center, single blind, randomized controlled Trial to prove that the BA9-DCB is non-inferior to the approved CE marked Sequent Please® Paclitaxel Coated Balloon with respect to % Diameter Stenosis and has similar safety characteristics. | Percent Diameter Stenosis (DS) [Time Frame: 6 months] assessed by QCA | **Experimental:** Biolimus A9™ Drug Coated Balloon  **Control:** Sequent ® Please Paclitaxel coated balloon | 201 |

CHA: Coronary Heart Disease; DCB: Drug-Coated Balloon; DEB: Drug Eluting Balloon; DES: Drug Eluting Stent; FDA: Food and Drug Administration; ISR: In-Stent Restenosis; LLL: Late Lumen Loss; MACE: Major Adverse Cardiovascular Events; MI: Myocardial Infarction; OCT: Optical Coherence Tomography; POBA: Plain Old Balloon Angioplasty; QCA: Quantitative Coronary Angiography; QFR: Quantitative Flow Ratio; SCAI: Society for Cardiovascular Angiography and Interventions; TLF: Target Lesion Failure; TLR: Target Lesion Revascularization; UDMI: Universal Definition of Myocardial Infarction.

# Supplemental Figures

## Supplemental Figure S1. Representative Core Lab OCT Analysis from the ISAR-DESIRE 5 Trial.

A Area: Lumen area; AS: Area stenosis; B Area: Stent Area; B Length: Visualized stent length; C Thickness: Neointimal thickness; DS: Diameter stenosis.





# Supplemental Methods

## Supplemental Methods S1. OCT-derived PCI-guidance

Decision-making should take into account the following aspects:

1. Morphology: in case of severe calcification (>180° of the circumference, >0.5mm thickness, >5mm in length) the use non-compliant balloon, intravascular lithotripsy, cutting/ scoring balloon, or atherectomy for lesion preparation;
2. Length-selection of the device: visually scan for the largest luminal area in the lumen profile proximally and distally, place landing zones in healthy tissue;
3. Diameter selection of the device: Take two external elastic laminae (EEL) measurements at both proximal and distal references to measure the vessel diameter. If two EEL measurements cannot be obtained, use lumen measurement. To choose the device diameter, use the distal reference measurements to select device diameter (EEL: round down to nearest stent size; lumen: round up to nearest stent size);
4. Post-dilation balloon selection (after stenting only): use the distal reference measurements from the baseline OCT to select post-dilation NC balloon diameter for the distal stent segment. Use the proximal reference measurement to select the post-dilation NC balloon diameter for the proximal stent segment. Use EEL and lumen as described previously.

## Supplemental Methods S2. Baseline clinical and interventional procedures performed according to standard clinical practice before randomization in the ISAR-DESIRE 5 Trial

1. In the context of this study, relevant medical history and physical examination, including pulse, blood pressure, height, and weight.
2. 12-lead ECG: includes at least type of rhythm, heart rate, and disturbances of repolarization.
3. Screening laboratory tests: white blood cell count, hemoglobin, hematocrit, platelet count, serum creatinine, glomerular filtration rate, activated partial thromboplastin time, international normalized ratio (INR), CK, CK-MB, troponin-T/I (if applicable high-sensitive), lipid status (HDL, LDL, total cholesterol), if applicable lipoprotein (a).
4. An oral loading dose of a P2Y12 inhibitor (Clopidogrel, Ticagrelor, or Prasugrel) will be given according to clinical presentation and current guidelines. All other medications such as nitrates, ß-blockers, etc. will be given at the discretion of the attending physician.
5. Diagnostic coronary angiography and left ventriculography will be performed according to local standard practice. The reference segment used to assess the severity of the stenosis will be defined as the average diameter of angiographically non-diseased proximal and distal segments adjacent to the lesion as the reference diameter. In cases where a long stent has been previously implanted, this average may include in-stent segments (provided they are not diseased).
6. OCT imaging of the target lesion will be performed per routine clinical practice and current recommendations**.**

## Supplemental Methods S3. Peri and post-interventional therapy in the ISAR-DESIRE 5 trial

Immediately after deciding to proceed with PCI, patients will be administered 500 mg of intravenous (i.v.) aspirin if they have not received it within the preceding 12 hours or are on chronic therapy. Additionally, patients will receive heparin via intra-arterial or i.v. routes, up to a total dose of 100 U/kg body weight, adjusting for any heparin received prior to the procedure. Alternatively, bivalirudin can be administered as an intravenous bolus of 0.75 mg/kg before the intervention starts, followed by an infusion of 1.75 mg/kg per hour throughout the procedure; local variations to this protocol are permissible. Glycoprotein IIb/IIIa inhibitors may be administered at the operator's discretion. Post-intervention, all patients will be prescribed aspirin indefinitely and a P2Y12 inhibitor (such as clopidogrel, prasugrel, or ticagrelor) at maintenance doses aligned with standard local practices and ESC guidelines for a duration of six months for patients with stable angina at admission, or 12 months for those with acute coronary syndrome. Patients on oral anticoagulation therapy, such as vitamin K antagonists or direct oral anticoagulants, will receive additional antiplatelet therapy tailored to local standards and in accordance with ESC guidelines.

## Supplemental Methods S4. Detailed definitions of qualitative OCT assessment

Adapted from Gonzalo et al.^24^

**Restenotic tissue structure:**

- **Homogenous:** Restenotic tissue has uniform optical properties and does not show focal variations in backscattering patterns.
- **Heterogeneous:** Restenotic tissue has focally changing in optical properties and shows various backscattering patterns.
- **Layered:** Restenotic tissue consists of concentric layers with different optical properties: an adluminal high scattering layer and an abluminal low scattering layer.

**Tissue backscatter patterns:**

- **High:** The majority of the tissue shows high backscatter and appears bright.
- **Low:** The majority of the tissue shows low backscatter and appears dark or black.

## Supplemental Methods S5. Endpoint’s definitions of the ISAR-DESIRE 5 trial

**Death**

*Classification of death:*

| **Type** | **Definition** |
| --- | --- |
| Cardiovascular death | Cardiovascular death is defined as death resulting from cardiovascular causes. The following categories may be collected:   1. Death caused by acute myocardial infarction 2. Death caused by sudden cardiac, including unwitnessed death 3. Death resulting from heart failure 4. Death caused by stroke 5. Death caused by cardiovascular procedures 6. Death resulting from cardiovascular hemorrhage 7. Death resulting from other cardiovascular cause |
| Non-cardiovascular death | Non-cardiovascular death is defined as any death that is not thought to be the result of a cardiovascular cause. The following categories may be collected:   1. Death resulting from malignancy 2. Death resulting from pulmonary causes 3. Death caused by infection (includes sepsis) 4. Death resulting from gastrointestinal causes 5. Death resulting from accident/trauma 6. Death caused by other non-cardiovascular organ failure 7. Death resulting from other non-cardiovascular cause |
| Undetermined death | Undetermined cause of death is defined as a death not attributable to any other category because of the absence of any relevant source documents. Such deaths will be classified as cardiovascular for end point determination. |

**Myocardial Infarction**

*Criteria for acute myocardial infarction:*

Detection of a rise and/or fall of cardiac troponin (cTn) with at least one value above the 99th percentile upper reference limit (URL) and with at least one of the following:

- Symptoms of ischemia;
- New ischemic ECG changes:
- Development of pathological Q waves in the ECG;
- Imaging evidence of new loss of viable myocardium or new regional wall motion abnormality in a pattern consistent with an ischemic etiology.
- Identification of a coronary thrombus by angiography or autopsy.

*Based on the Fourth Universal Definition, myocardial infarction will be classified into various types:*

| **Type** | **Constellation** | **Definition** |
| --- | --- | --- |
| Type 1 | Spontaneous myocardial infarction | MI caused by atherothrombotic CAD and usually precipitated by atherosclerotic plaque disruption (rupture or erosion) |
| Type 2 | Myocardial infarction secondary to an ischemic imbalance | In instances of myocardial injury where a condition other than CAD contributes to an imbalance between myocardial oxygen supply and/or demand, e.g. coronary endothelial dysfunction, coronary artery spasm, coronary embolism, tachy-/brady-arrhythmias, anemia, respiratory failure, hypotension, and hypertension with or without LVH. |
| Type 3 | Myocardial infarction resulting in death when biomarker values are unavailable | Patients who suffer cardiac death, with symptoms suggestive of myocardial ischemia accompanied by presumed new ischemic ECG changes or ventricular fibrillation, but die before blood samples for biomarkers can be obtained or before increases in cardiac biomarkers can be identified or MI is detected by autopsy examination. |
| Type 4a | Myocardial infarction related to PCI | Myocardial infarction associated with PCI is arbitrarily defined by elevation of cTn values > 5 x 99th percentile URL in patients with normal baseline values (< 99th percentile URL) or a rise of cTn values > 20% if the baseline values are elevated and are stable or falling. In addition, either   1. new ischemic ECG changes, or 2. angiographic findings consistent with a procedural flow-limiting complication or 3. imaging demonstration of new loss of viable myocardium or new regional wall motion abnormality are required |
| Type 4b | Myocardial infarction related to stent thrombosis | A subcategory of PCI-related MI is stent thrombosis, type 4b MI, as documented by angiography or autopsy using the same criteria utilized for type 1 MI |
| Type 4c | Myocardial infarction related to in-stent restenosis | This PCI-related MI type is defined as focal or diffuse restenosis, or a complex lesion associated with a rise and/or fall of cTn values above the 99th percentile URL applying, the same criteria utilized for type 1 MI. |
| Type 5 | Myocardial infarction related to coronary artery bypass grafting (CABG) | Myocardial infarction associated with CABG is arbitrarily defined by elevation of cardiac biomarker values > 10 x 99th percentile URL in patients with normal baseline cTn values. In patients with elevated pre-procedure cTn in whom cTn levels are stable (≤ 20% variation) or falling, the postprocedure cTn must rise by > 20%. In addition, either   1. new pathological Q waves, or 2. angiographic documented new graft or new native coronary artery occlusion, or 3. imaging evidence of new loss of viable myocardium or new regional wall motion abnormality. |

**Procedure-Related Myocardial Infarction**

*For the protocol-relevant definition of procedure-related myocardial infarction, patients must fulfill the following criteria 1 AND 2 OR 1 AND 3 AND 4.*

| **Constellation** | **Definition** |
| --- | --- |
| Patients without NSTEMI | 1. any CK-MB (or CK) ≥3 ULN   AND   1. elevation at least 50 % over the most recent pre-PCI levels   OR   1. new ECG changes consistent with MI    1. New or re-elevation of ST-segments ≥0.2mV (0.08sec after the J-point) in ≥2 contiguous precordial leads or ≥0.1 mV in ≥2 adjacent limb ECG leads. *(ST-depression in V1-V3 will be considered equivalent to ST- elevation if the recurrent MI is suspected to be true posterior)*  OR    2. Development of new, abnormal Q-waves (≥0.04sec in duration are present in 2 contiguous precordial leads or 2 adjacent limb leads). *(An increase in R wave amplitude in V1-V3 will be considered equivalent to Q-waves if the recurrent MI is suspected to be true posterior)*   AND   1. CK-MB (CK) higher than the upper normal limit at two measurements. |
| Patients with NSTEMI and CK-MB (CK) levels are falling or normal | 1. Any CK-MB (or CK) ≥3 ULN   AND   1. elevation at least 50 % over the most recent pre-PCI levels   OR   1. new ECG changes consistent with MI    1. New or re-elevation of ST-segments ≥0.2mV (0.08sec after the J-point) in ≥2 contiguous precordial leads or ≥0.1 mV in ≥ 2 adjacent limb ECG leads. *(ST-depression in V1-V3 will be considered equivalent to ST- elevation if the recurrent MI is suspected to be true posterior)* OR    2. Development of new, abnormal Q-waves (≥0.04sec in duration are present in 2 contiguous precordial leads or 2 adjacent limb leads). *(An increase in R wave amplitude in V1-V3 will be considered equivalent to Q-waves if the recurrent MI is suspected to be true posterior)*   AND   1. CK-MB (CK) higher than the upper normal limit at two measurements. |
| Patients with NSTEMI, where peak CK-MB (CK) level have not yet been reached before PCI | 1. recurrent chest pain ≥ 30 minutes   OR   1. new ECG changes consistent with second MI New or re-elevation of ST-segments ≥0.2 mV (0.08sec after the J-point) in ≥2 contiguous precordial leads or ≥0.1 mV in ≥2 adjacent limb ECG leads. *(ST-depression in V1-V3 will be considered equivalent to ST- elevation if the recurrent MI is suspected to be true posterior)*   AND   1. next CK-MB (CK) level at 8-12h after PCI is elevated at least 50 % above the previous level   OR   1. Development of new, abnormal Q-waves (≥0.04sec in duration are present in 2 contiguous precordial leads or 2 adjacent limb leads).   *(An increase in R wave amplitude in V1-V3 will be considered equivalent to Q-waves if the recurrent MI is suspected to be true posterior)* |

In addition, the SCAI-definition of procedure-related myocardial infarction may be considered:

| Patients with normal baseline CK-MB | The peak CK-MB measured within 48 hours of the procedure rises to ≥10 × the local laboratory ULN, or to ≥5 × ULN with new pathologic Q-waves in ≥2 contiguous leads or new persistent LBBB, OR in the absence of CK-MB measurements and a normal baseline cTn, a cTn (I or T) level measured within 48 hours of the PCI rises to ≥70 × the local laboratory ULN, or ≥35 ×ULN with new pathologic Q-waves in ≥2 contiguous leads or new persistent LBBB. |
| --- | --- |
| Patients with elevated baseline CK-MB (or cTn) in whom the biomarker levels are stable or falling | The CK-MB (or cTn) rises by an absolute increment equal to those levels recommended above from the most recent pre-procedure level. |
| Patients with elevated CK-MB (or troponin) in whom the biomarker levels have not been shown to be stable or falling | The CK-MB (or cTn) rises by an absolute increment equal to those levels recommended above plus new ST-segment elevation or depression plus signs consistent with a clinically relevant MI, such as new onset or worsening heart failure or sustained hypotension. |

**Q-wave vs. Non-Q-wave Myocardial infarction**

Q-wave MI will be diagnosed if new pathologic Q-waves (≥25% of the height of the partner R wave and/or ≥0.04 sec in duration) in ≥2 contiguous ECG leads occur. All other myocardial infarctions not fulfilling the above-mentioned criteria will be considered non-Q-wave myocardial infarctions.

| **Type** | **Definition** |
| --- | --- |
| Definite | Angiographic confirmation of stent thrombosis: The presence of a thrombus that originates in the stent or in the segment 5 mm proximal or distal to the stent / scaffold or in a side branch originating from the stented segment and the presence of at least 1 of the following criteria:  Acute onset of ischemic symptoms at rest:   - New electrocardiographic changes suggestive of acute ischemia - Typical rise and fall in cardiac biomarkers (refer to definition of spontaneous myocardial infarction)   OR  Pathological confirmation of stent thrombosis:   - Evidence of recent thrombus within the stent/scaffold determined at autopsy - Examination of tissue retrieved following thrombectomy (visual/histology) |
| Probable | Regardless of the time after the index procedure, any myocardial infarction that is related to documented acute ischemia in the territory of the implanted stent without angiographic confirmation of stent thrombosis and in the absence of any other obvious cause. |
| Silent | The incidental angiographic documentation of stent occlusion in the absence of clinical signs or symptoms is not considered stent thrombosis. |
| **Timing** | |
| Acute | ≤24 hours following the index PCI |
| Subacute | >24 hours to ≤30 days after the index PCI |
| Late | >30 days to ≤1 year following the index PCI |
| Very late | >1 year following the index PCI |

**Stent Thrombosis**

**Target Lesion Revascularization**

The target lesion is defined as the treated segment, including the 5-mm margin proximal and distal to the stent. Target lesion revascularization is defined as a repeat percutaneous intervention of the target lesion or bypass surgery of the target vessel performed for restenosis or other complications of the target lesion. Ischemia-driven target lesion revascularization is considered in the following cases:

- Diameter stenosis ≥50 % (“in-segment” QCA-analysis) at follow-up angiography and the patient had a positive functional study corresponding to the area served by the target lesion, and ischemic symptoms and ECG-changes at rest referable to the target lesion.
- Diameter stenosis <50 % at follow-up angiography but a markedly positive functional study or ECG-changes corresponding to the territory supplied by target vessel.
- Diameter stenosis ≥70 % at follow-up angiography in absence of documented clinical or functional ischemia.

**Target Vessel Revascularization**

The target vessel is defined as the entire major intervened coronary vessel, including side branches. Target vessel revascularization is defined as any repeat percutaneous intervention or surgical bypass of any segment of the target vessel including the target lesion. Ischemia-driven target vessel revascularization is considered in the following cases:

- Diameter stenosis ≥50 % (“in-segment” QCA-analysis) at follow-up angiography and the patient had a positive functional study corresponding to the area served by the target lesion, and ischemic symptoms and ECG-changes at rest referable to the target lesion.
- Diameter stenosis <50 % at follow-up angiography but a markedly positive functional study or ECG-changes corresponding to the territory supplied by target vessel.
- Diameter stenosis ≥70 % at follow-up angiography in absence of documented clinical or functional ischemia.

**Urgent Revascularization**

Any PCI or bypass surgery for recurrent ischemia that cannot be delayed for more than 24 hours in the investigator’s opinion and that is defined as a non-elective procedure by the investigator.
